# The Janus face of DNA methylation in aging

**DOI:** 10.18632/aging.100124

**Published:** 2010-03-03

**Authors:** Chris Mugatroyd, Yonghe Wu, Yvonne Bockmühl, Dietmar Spengler

**Affiliations:** Max Planck Institute of Psychiatry, Molecular Neuroendocrinology, D-80804 Munich, Germany

**Keywords:** Methylation, aging, early-life adversity, vasopressin, MeCP2

## Abstract

Aging is arguably the most familiar yet least-well understood aspect of human biology. The role of epigenetics in aging and age-related diseases has gained interest given recent advances in the understanding of how epigenetic mechanisms mediate the interactions between the environment and the genetic blueprint. While current concepts generally view global deteriorations of epigenetic marks to insidiously impair cellular and molecular functions, an active role for epigenetic changes in aging has so far received little attention. In this regard, we have recently shown that early-life adversity induced specific changes in DNA methylation that were protected from an age-associated erasure and correlated with a phenotype well-known to increase the risk for age-related mental disorders. This finding strengthens the idea that DNA (de-)methylation is controlled by multiple mechanisms that might fulfill different, and partly contrasting, roles in the aging process.

Although age is by far the biggest risk factor for a wide range of clinical conditions that are prevalent today, old-age survival has increased substantially during the past half century. Baby boom generations are growing older, the chance of surviving to old age is increasing, and the elderly are living longer due to remarkable, though largely unexplained, reductions in mortality at older ages [1]. Not surprisingly, these puzzling biodemographic trajectories are difficult to reconcile with present theories about aging. A key assumption underlying the theory of evolution holds that fertility and survival schedules are fixed − a questionable premise for most species in the wild that have evolved alternate physiological modes for coping with fluctuating environmental conditions including dauer states (*C. elegans*), stationary phase (yeast), diapause (certain insects) and hibernation. Furthermore, studies in social insects, particularly the honey-bee, have revealed that the same genome can be alternatively programmed to produce short-lived workers or long-lived queens. By and by we are coming to realize  that the evolution of whole organisms' traits (birth sizes, growth rates, age and size at maturity, reproductive investment, mortality rates and lifespan) is crucially shaped by the interaction of intrinsic and extrinsic factors. How the genetic blueprint and environmental influences interact with each other is of utmost interest especially in aging research. Many lines of evidence, including large epidemiological and extensive clinical and experimental studies, support the notion that early life events strongly influence later susceptibility to chronic diseases and mortality rates. An increased understanding of the ability of an organism to develop in various ways (developmental plasticity), depending on a particular environment or settings, provides a conceptual basis for these observations and current biodemographic trends [2, 3].

Developmental plasticity requires stable modulation of gene expression, and this appears to be mediated, at least in part, by epigenetic processes such as DNA methylation and histone modification. This concept entails, however, the question of whether those epigenetic marks relate to age-associated declines in molecular and cellular functions. Indeed, the current literature favors the view that epigenetic mechanisms such as DNA methylation deteriorate with age and may even accelerate the aging process [4]. A relationship between DNA methylation and aging was originally proposed in a pioneering study by Berdyshev [5], which showed that genomic global DNA methylation decreases with age in spawning humpbacked salmon. In support of this finding, a gradual global loss of cytosine methylation has been detected in various mouse, rat and human tissues [6, 7, 8]. Moreover, different types of interspersed repetitive sequences, which make up a major fraction of mammalian genomes, appear to be targeted at different ages and to varying degrees by age-associated hypomethylation [9]. This finding is compatible with the presence of several mechanisms that regulate global hypomethylation and possibly contribute at different steps to the aging process.

**Figure 1.**
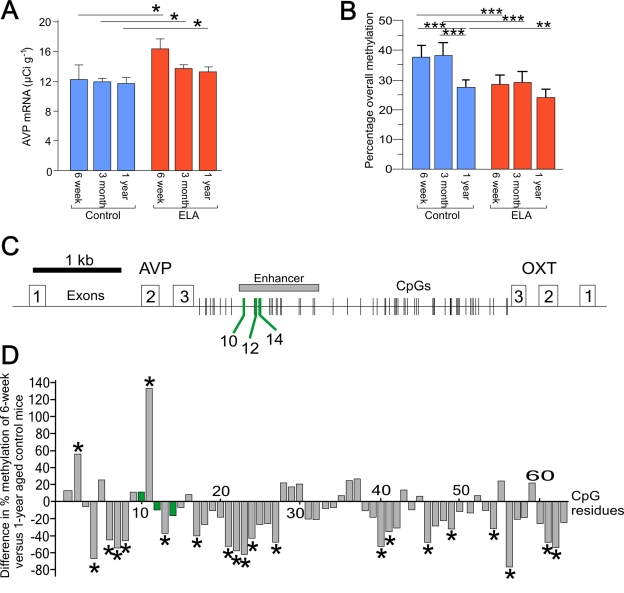
Age related changes in AVP expression and DNA methylation. **(A)** Aging does not affect AVP mRNA expression in control mice. Early-life adversity (ELA) leads to a persistent increase in AVP mRNA expression. *P < 0.05. **(B)** Age-dependent hypo-methylation occurs only in the control mice. Early-life adversity leads to a persistent hypomethylation across the enhancer region in 6-week old mice. **P < 0.005 and ***P < 0.0001. **(C)** Schematic diagram of the *AVP* and *oxytocin* genes orientated tail-to-tail and separated by the intergenic region (IGR). Exons are indicated by open numbered boxes and distribution of CpG residues is shown. The downstream enhancer is boxed in gray with MeCP2 DNA-binding sites (CpG10, 12, and 14) indicated by green lines. **(D) **Comparison of the methylation status of all CpGs in the IGR between 6-week and 1-year aged control mice shows that the majority of CpGs in the control mice undergo hypomethylation. In contrast, those methylation landmarks mapping to MeCP2 DNA-binding sites (marked in green) are protected from age-associated changes in DNA methylation.

Aside from global hypomethylation, a number of specific loci have been reported to become hypermethylated during aging (the ribosomal gene cluster, the estrogen receptor, insulin growth factor, E-cadherin, c-fos etc.; reviewed in [10]). In general terms, age-associated hypermethylation is thought to preferentially affect loci at CpG islands, while loci devoid of CpG islands loose methylation with age. In addition, a study in humans has revealed that intra-individual changes in DNA methylation show some degree of familial clustering, indicative of a genetic component [11].

Taken together, these results seem to imply that early-life induced programming − in so far that it relies on DNA methylation − is at a considerable risk to become insidiously disrupted during aging. This erasing might curtail any long-lasting programs derived from early-life conditions.

A recent study in mice sheds new light on this topic. Murgatroyd and coworkers [12] showed that early-life adversity (daily 3-hour separation of mouse pups from their mother during postnatal days 1−10) caused persistent hypomethylation at a discrete region of the arginine vasopressin (*AVP*) gene enhancer (Figure 1C) in the hypothalamic nucleus paraventricularis (PVN). This led to a sustained overexpression of *AVP* (Figure 1A), a key activator of the hypothalamo-pituitary adrenal (HPA) stress axis. As a result, early-life adversity evoked a lifelong elevation in glucocorticoid (GC) secretion, heightened endocrine responsiveness to stressors, reduced stress coping ability and memory deficits. All of these neuroendocrine and behavioral alterations are well-known risk factors for aging and frequent features of age-associated brain pathologies such as major depression and dementia (for review [13, 14]).

The *AVP* enhancer is located downstream of the *AVP* gene in the intergenic region (IGR) separating the *AVP* and *oxytocin* genes (Figure 1C). Analysis of overall CpG methylation across the *AVP* enhancer revealed that the early-life adversity-induced hypomethylation was strongest at 6 weeks of age though less prominent in 1-year aged mice compared to controls (Figure 1B). In contrast, control mice alone showed a clear decrease in methylation at 1 year of age even though AVP mRNA levels remained unaltered (Figure 1A). This finding suggests that early-life adversity-induced hypomethylation correlated functionally with increased *AVP* transcription and persisted over time, while age-associated hypomethylation of the *AVP* enhancer in control mice lacked per se a functional correlate. This puzzling constellation led the authors to hypothesize that single CpG residues at the *AVP* enhancer behaved differentially with respect to early-life versus age-associated hypomethylation. To elucidate the cause of such functional heterogeneity among CpG residues at the *AVP* enhancer, they went on to correlate CpG methylation across the entire enhancer with transcriptional activity of the *AVP* gene. This allowed the identification of a number of CpG residues (CpG10 and CpGs 12-15 dubbed 'methylation landmarks') that strongly correlated with AVP transcription affinity DNA-binding sites of the epigenetic reader and writer MeCP2 (methyl-CpG-binding protein 2)(Figure 2C). MeCP2 serves as a platform upon which synergistic crosstalk between histone deacetylation, H3K9 methylation and DNA methylation is played out to confer transcriptional repression and gene silencing (for an in depth discussion of MeCP2's role in AVP regulation see [15]).

A comparison of the methylation status of all CpG residues in the IGR in 6-week and 1-year aged control mice showed that those CpG residues mapping to MeCP2 DNA-binding sites (marked in green) did not change in the degree of their methylation (Figure 1D). In contrast, 30% of the remaining CpG residues underwent a significant age-related hypomethylation, while only very few CpG residues (3%) showed a significant increase. As noted before, this age-associated hypomethylation did not trigger *per se* enhanced *AVP* gene expression.

**Figure 2.**
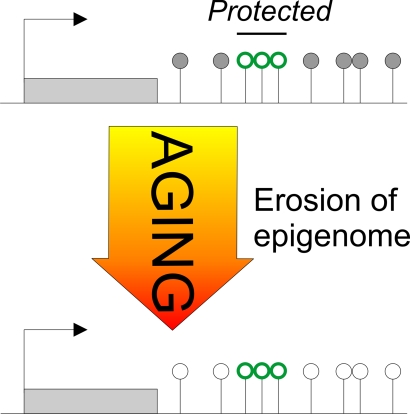
The Janus face of DNA methylation in aging. Early-life adversity-induced hypomethylation centers on CpG residues mapping to DNA-binding sites of the epigenetic reader and writer MeCP2 (red lollipops). Once established, these methylation landmarks are maintained and do not undergo further age-associated changes in methylation. In contrast, age-associated hypomethylation maps across the entire AVP locus without any obvious pattern or preference for potential DNA-binding sites (black and white lollipops). In this regard, age-associated hypomethylation appears to behave stochastically, while early-life adversity is targeted.

Taken together, AVP exemplifies an unexpected double-faced role of DNA methylation in aging. Hereby, specific environmental stimuli (such as early-life adversity) can induce site-specific changes in DNA methylation at critical regulatory sites that underpin sustained changes in gene expression subsequently influencing the risk of age-associated pathologies. These epigenetic changes are actively controlled and couple to specific stimuli targeting distinct genes. Due to active maintenance mechanisms (albeit this does not exclude their extinction by compensatory or counteracting processes) these epigenetic marks are largely protected from age-associated changes in DNA methylation (Figure 2).

It appears that age-associated genome-wide and site-specific (de-)methylation can indistinguishably disrupt gene expression profiles and lead to the deterioration of cellular functions. These processes seem to be independent of a specific stimulus during a critical time window and take place in multiple, unrelated species. Despite some preliminary evidence from humans that structural criteria of the DNA (CpG island or the type of repetitive element) age-associated changes in methylation remain enigmatic. Importantly, however, age-associated changes in methylation do not inevitably override early life-induced epigenetic programming (in fact, age-associated hypomethylation of the *AVP* enhancer had no effect on mRNA expression levels) and strengthen the idea that these two processes are functionally and mechanistically distinct. Further research will be needed to substantiate this concept. However, current work on epigenetic programming of mice does suggest that differential changes in methylation in response to early-life adversity and aging apply to other genes in addition to *AVP* (Y. Wu, unpublished data). Certainly, the advancement of genome-wide approaches [16] combining high resolution analysis and functional studies in the field of epigenetics has the potential to accelerate dramatically our understanding of the underlying mechanisms in aging and age-associated diseases, ultimately opening up new possibilities in diagnosis and treatment.
